# Supplementary material for: Autofluorescence imaging permits label-free cell type assignment and reveals the dynamic formation of airway secretory cell associated antigen passages (SAPs)
Source: eLife. 2023 Mar 30;12:e84375. doi: 10.7554/eLife.84375 (PMC10154029; doi:10.7554/eLife.84375)
Supplement: Figure 4—source data 1. [file elife-84375-fig4-data1.docx]

|  | Secretory Cells with Voids | Secretory Cells without voids |
| --- | --- | --- |
| Baseline | 17 | 181 |
| 30 mins with Methacholine | 155 | 43 |

**Figure 4 Source Data 1. Secretory cells with “voids” are present at baseline and their numbers increase following 30 minutes of methacholine treatment.** Quantification of “voids” in secretory cells (identified by autofluorescence) within 26051µm^2^ of a tracheal surface (from Figure 4C) at baseline and after the application of 10µM methacholine for 30 minutes.
